# The Interplay between Light Quality and Biostimulant Application Affects the Antioxidant Capacity and Photosynthetic Traits of Soybean (*Glycine max* L. Merrill)

**DOI:** 10.3390/plants10050861

**Published:** 2021-04-24

**Authors:** Ermenegilda Vitale, Violeta Velikova, Tsonko Tsonev, Ida Ferrandino, Teresa Capriello, Carmen Arena

**Affiliations:** 1 Department of Biology, University of Naples Federico II, Via Cinthia 26, 80126 Naples, Italy; ermenegilda.vitale@unina.it (E.V.); ida.ferrandino@unina.it (I.F.); teresa.capriello@unina.it (T.C.); 2 Institute of Plant Physiology and Genetics, Bulgarian Academy of Sciences, Acad. G. Bonchev Street bl. 21, 1113 Sofia, Bulgaria; 3 Institute of Biophysics and Biomedical Engineering, Bulgarian Academy of Sciences, Acad. G. Bonchev Street bl. 21, 1113 Sofia, Bulgaria; ttsonev@bio21.bas.bg; 4 BAT Center-Interuniversity Center for Studies on Bioinspired Agro-Environmental Technology, 80055 Portici, Italy

**Keywords:** sprout bioactive compounds, light quality modulation, amino acids based biostimulant, PSII photochemical efficiency

## Abstract

This paper evaluates the combined effect of biostimulant and light quality on bioactive compound production and seedling growth of soybean (*Glycine max* L. Merrill) plants. Germinated seeds pre-treated with different concentrations (0.01%, 0.05%, 0.5%) of an amino acid-based biostimulant were grown for 4 days at the dark (D), white fluorescent light (FL), full-spectrum LED (FS), and red-blue (RB) light. Potential changes in the antioxidant content of sprouts were evaluated. Part of the sprouts was left to grow at FL, FS, and RB light regimes for 24 days to assess modifications in plants’ anatomical and physiological traits during the early developmental plant stage. The seed pre-treatment with all biostimulant concentrations significantly increased sprout antioxidant compounds, sugar, and protein content compared to the control (seeds treated with H_2_O). The positive effect on bioactive compounds was improved under FS and RB compared to D and FL light regimes. At the seedling stage, 0.05% was the only concentration of biostimulant effective in increasing the specific leaf area (SLA) and photosynthetic efficiency. Compared to FL, the growth under FS and RB light regimes significantly enhanced the beneficial effect of 0.05% on SLA and photosynthesis. This concentration led to leaf thickness increase and shoot/root ratio reduction. Our findings demonstrated that seed pre-treatment with proper biostimulant concentration in combination with specific light regimes during plant development may represent a useful means to modify the bioactive compound amount and leaf structural and photosynthetic traits.

## 1. Introduction

In the last decades, agricultural practices are changing to meet the increasing market demand in response to the nutritional requirements of a growing population. This high production of food is leading to an overexploitation of the resources, especially of the soil, also exacerbated by climate changes [1]. In this context, new cultivation techniques in agriculture that minimize environmental impacts and cope with the lack of resource availability should be desirable as well as the possibility to produce functional food out of the soil or on suitable substrates.

Light modulation in terms of quality, intensity, and duration deeply influences plant morphogenesis, photosynthesis, and growth. Currently, the manipulation of the light quality as a tool to obtain specific physiological and morphological traits is largely used in controlled environment agriculture (CEA), an innovative approach in which crops are cultivated indoor (greenhouses, growth chambers, vertical farms) to optimise their cultivation for food, pharmaceutical, and nutraceutical applications and save resources [2,3]. The light modulation approach allows an in-depth understanding of the photosynthetic responses to different light wavelengths [4,5] as well as the plant potentiality to produce bioactive compounds induced by diverse light quality treatments [6]. Several studies have been carried out on different crops, testing monochromatic light or mixing different wavelengths. Bian et al. [7] reported that the accumulation of phytochemicals in vegetable crops, such as lettuce, cucumber, tomato, radish, and spinach, depended on light quality and intensity. Light quality affects mainly carotenoids, phenolics, and vitamin C [8]. Among the visible spectra, red and blue lights are essential for photosynthesis and have often been used in plant research and for commercial production. It was previously reported that red and blue LEDs effectively enhance plant growth and secondary metabolites synthesis, and these effects are species-specific [9,10,11]. Despite the contrasting results, the primary outcomes agree that red and blue wavelengths are absorbed in the top of the leaves/canopy and are the most used regions of the light spectrum driving the photosynthetic process, biomass accumulation, shoot elongation, root development, stomata opening/closing regulation mechanism, pigment and polyphenol synthesis [7,12,13,14,15]. However, the green component penetrating deeper in the leaf tissues and canopy layer promotes the CO_2_ fixation in regions not sufficiently reached by blue and red lights [16]. Overall, the choice of specific wavelengths matching plant photoreceptors can determine plant morphology, physiology, and metabolism, allowing us to define suitable light fertilization protocols [17].

As light quality modulation, the application of biostimulants may also be considered as an innovative eco-friendly and promising strategy replacing the common chemical fertilizers [18,19]. Biostimulants of different origins exist, including bacteria, fungi, seaweeds, higher plant extracts, protein hydrolysates (PHs) [20]. The composition, as well as the application strategies (at seed, soil, or leaf level), may influence their mode of action and the effects on crops. Several classes of biostimulants are highly used to improve seed germination, root system development, nutrient absorption, growth, productivity, and tolerance to environmental stresses [21,22,23]. Currently, the use of biostimulants in agriculture is increasing due to the need for low impact and more sustainable agricultural management approaches [24]. Among available classes, the biostimulants based on protein hydrolysates and the products containing amino acids are particularly worthy of attention because they enhance plant yield and quality in terms of growth, phytochemical content, N-uptake, and tolerance to many abiotic stresses [21,23,25,26]. Recent researches have specifically demonstrated that biostimulants can improve the nutritional traits of some plant-derived foods by enhancing the accumulation of secondary metabolites and phytonutrients in different parts of the plant [27].

Based on experimental evidence, the biostimulants application as well as the light quality manipulation are key aspects to be addressed in the next years for sustainable agricultural management approaches. Nowadays, only a few studies investigated the joined effect of light spectrum modulation and biostimulant showing responses depending on species and its phenotypic plasticity [19,28]. This paper aimed to explore the potential beneficial effects of the biostimulant application under different light quality regimes on plant bioactive compounds, seedling development, and photosynthesis. Soybean (*Glycine max* L. Merrill) was selected as a model species as it is largely desired in the marketplace for the high nutritional properties of seeds and sprouts [29]. In the present work, we treated soybean seeds with increasing doses of a new amino acid-based biostimulant (B) and tested the best concentration for promoting seed germination and bioactive compound synthesis in sprouts. Thereafter, the biostimulant pre-treated seeds were exposed to specific light quality (LQ) regimes (white fluorescent, FL; full-spectrum, FS, and red-blue, RB) to assess if the interaction biostimulant × light quality (B × LQ) may enhance the sprout nutritional value and photosynthetic activity of seedlings during the early developmental stage improving the overall seedling growth performance.

The outcomes of this study may be useful for the development of new protocols for the cultivation of soybean on a broad scale in the context of sustainable agriculture and to improve soybean sprout quality.

## 2. Results

### 2.1. Effect of Biostimulant on Seed Germination

One-way ANOVA analysis demonstrated that different concentrations (0.01, 0.05 and 0.5%) of the Kaishi biostimulant (K-) did not affect neither the germination percentage (G%) nor days to 50% emergence (E_50_) compared to control (treated with H_2_O). The G% values were 86 ± 4.4^a^, 80 ± 5.0^a^, 83 ± 3.3^a^, 83 ± 6.2^a^ for H_2_O, K-0.01%, K-0.05% and K-0.5%, respectively. The observed E_50_ values were: 1.9 ± 0.3^a^, 2.0 ± 0.1^a^, 2.1 ± 0.2^a^, 2.0 ± 0.4^a^ for H_2_O, K-0.01%, K-0.05% and K-0.5%, respectively.

### 2.2. Effect of Biostimulant and Light Regimes on Sprout Bioactive Compounds, Proteins, and Sugars

**Figure 1:**
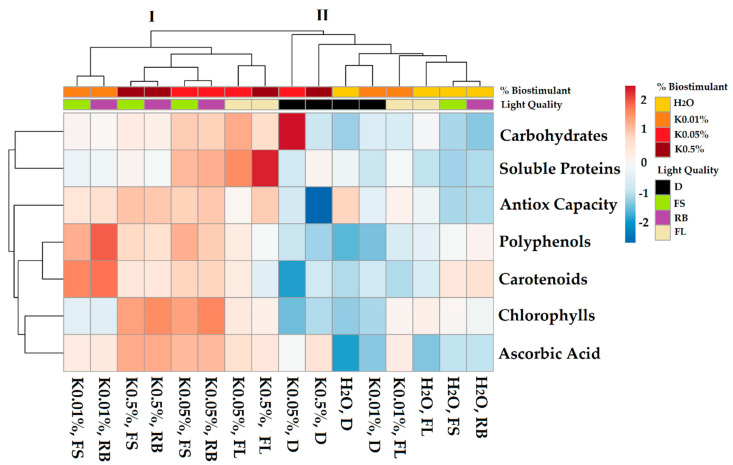
Cluster heatmap analysis summarizing qualitative traits of soybean sprouts (8 DAS) in response to different concentrations of biostimulant (K-0.01, K-0.05, and K-0.5%) and different light quality regimes (dark-D, white fluorescent-FL, full-spectrum-FS and red-blue-RB). Seeds treated with H_2_O served as a control. Numeric differences within the data matrix are shown by the color scale: red and blue indicate increasing and decreasing values, respectively. Parameters are clustered in the rows; sample groups are clustered in the columns by the two independent factors Biostimulant and Light Quality.

Figure 1 shows an overview of the qualitative traits of soybean sprouts in response to different biostimulant concentrations (K-0.01, K-0.05, and K-0.5%) and light quality regimes (dark, FL, FS, and RB).

The heatmap established two main clusters (I and II), which strongly depended on the applied B and LQ regimes. Cluster II included all sprouts grown under dark, all control (H_2_O irrespective of the light regime), and K-0.01% × FL sprouts. Conversely, cluster I incorporated the remaining part of the testing groups. Cluster I showed higher values of biochemical compounds compared to cluster II. In particular, within cluster I, the subcluster composed of K-0.05% × FS and K-0.05% × RB sprouts, was characterized by a higher level of nutraceutical traits.

The effects of biostimulant and light quality as independent factors and their interaction on bioactive compounds of soybean sprouts were reported in Table 1.

The content of the bioactive compounds in soybean sprouts was influenced by B and LQ as main factors and their interaction (B × LQ). The only exception was the total carotenoid content, which was not affected by B, and carbohydrate amount was not affected by different LQ (Table 1). Regardless of the LQ regimes, among B treatments, K-0.05% and K-0.5% increased (*p* < 0.001) the chlorophyll content (Table 1). An increase in soluble protein level (*p* < 0.001) was found only in sprouts pre-treated with K-0.01% and K-0.05% showing the highest value at K-0.05% (Table 1). Compared to control, all B concentrations promoted (*p* < 0.001) ascorbic acid and total polyphenols content, as well as the antioxidant capacity (*p* < 0.01) and carbohydrates (*p* < 0.01) amount. In particular, carbohydrates reached the highest value (*p* < 0.001) at K-0.05% (Table 1).

Compared to sprouts exposed to darkness (D), those developed under FL, FS, and RB light regimes displayed greater (*p* < 0.001) antioxidant capacity, polyphenol, chlorophyll, carotenoid, ascorbic acid, and protein content independently from the biostimulant concentration (Table 1). In particular, the total polyphenols, chlorophylls, carotenoids, and ascorbic acid reached the highest values (*p* < 0.001) under FS and RB compared to FL light regime, which showed the highest (*p* < 0.001) protein concentration (Table 1).

As regards the interaction, all combinations B × LQ were significant (Table 1). In particular, K-0.01/0.05/0.5% × RB and K-0.01/0.05/0.5% × FS promoted TAC compared to H_2_O × RB and FS. Conversely, K-0.01/0.05/0.5% × D reduced TAC compared to H_2_O × D. The combinations K-0.05% × FS and K-0.05/0.5% × RB significantly increased CHL. Among all interactions, K-0.01% × RB induced the greatest TPC, while K-0.05% × D the highest CARB value. The interaction of K-0.05 and K-0.5% × FS and RB produced the highest AsA content, while K-0.5% × FL was the most effective in increasing the SP content.

### 2.3. Influence of Biostimulant and Light Quality on Seedling Morpho-Anatomical and Physiological Parameters

**Figure 2:**
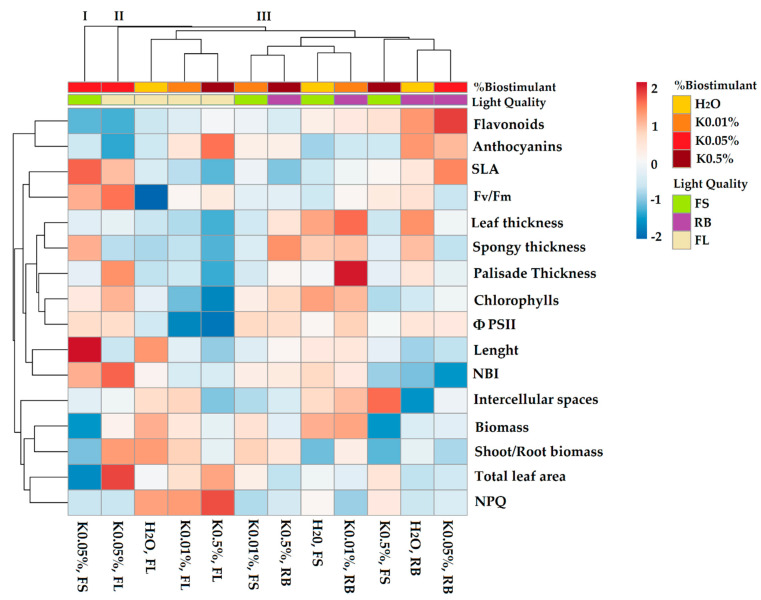
Cluster heatmap analysis summarizing physiological and morpho-anatomical parameters of soybean seedlings (at 24 DAS) in response to different concentrations of biostimulant (K-0.01, K-0.05, and K-0.5%) and different light quality regimes (white fluorescent-FL, full-spectrum-FS and red-blue-RB). Seeds treated with H_2_O served as a control. Numeric differences within the data matrix are shown by the colour scale: red and blue indicate increasing and decreasing values, respectively. Parameters are clustered in the rows; sample groups are clustered in the columns by the two independent factors, biostimulant and light quality.

Figure 2 summarises the physiological and morphological traits of soybean seedlings in response to different B concentrations (K-0.01, K-0.05, and K-0.5%) and LQ regimes (FL, FS, and RB).

The heatmap established three main clusters. The first cluster (I) included K-0.05% × FS seedlings, while the second (II) only contained K-0.05% × FL seedlings. The third cluster (III) was divided into two subclusters. One, on the left, included all the seedlings developed under FL light (H_2_O, K-0.01, K-0.5%). The second, on the right, incorporated the other testing groups. The generation of clusters I and II identified the B as the main discriminant factor compared to LQ, suggesting that the concentration K-0.05% significantly affected both soybean physiological and morphological traits. Within cluster III, LQ acted as the main discriminant factor compared to the biostimulant application and separated FL from FS and RB seedlings. Within the FL group, the separation of control from biostimulant-treated seedlings was evident. Conversely, within the second subcluster, no clear division between FS and RB seedlings occurred. Cluster I was characterised by higher values of SLA, photochemical PSII efficiency, NBI, plant length, total leaf area. Cluster II displayed a higher shoot/root biomass ratio and NPQ. Cluster III grouped seedlings with elevated values of leaf thickness (spongy and palisade), intercellular spaces, and pigment content.

#### 2.3.1. Morphological Traits and Leaf Anatomy

Analysis of variance revealed that different B concentrations did not affect as main factors total plant leaf area, plant length, total plant biomass, and shoot/root biomass allocation, but significantly (*p* < 0.001) modified SLA (Table 2). Conversely, LQ influenced the morphological leaf traits and biomass partitioning. In particular, FS and RB seedlings showed a reduced (*p* < 0.05) leaf area and shoot/root biomass allocation (*p* < 0.001) and an increase in SLA (*p* < 0.05) (Table 2) when compared to FL. No significant interaction B × LQ was found in morphological parameters except for SLA (*p* < 0.05) (Table 2).

The interactions K-0.05% × FL, K-0.05% × FS, and K-0.05% × RB induced the highest (*p* < 0.001) SLA values (Table 2).

**Figure 3:**
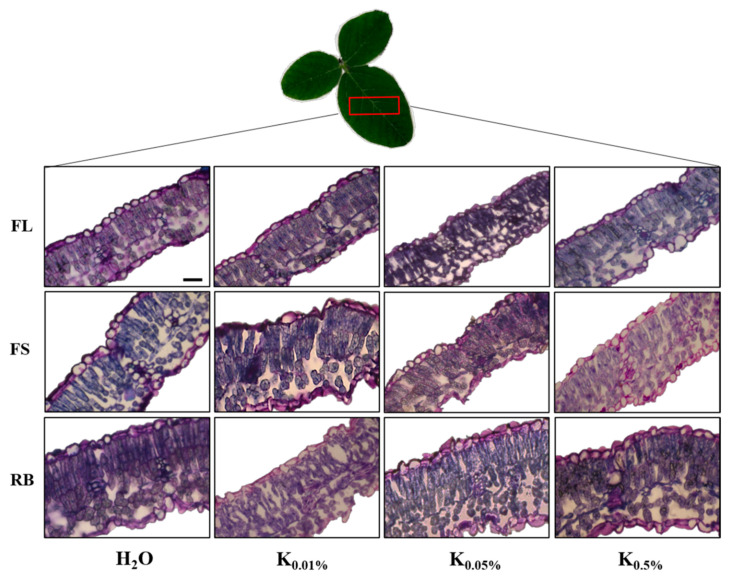
Cross-sections (stained by Toluidine blue) of leaf lamina of soybean seedlings at 24 DAS sprouted from seeds pre-treated with different concentrations of biostimulant Kaishi (K-0.01, K-0.05, and K-0.5%) and grown under different light quality regimes: white fluorescent-FL, full-spectrum-FS and red-blue-RB. Seeds treated with H_2_O served as a control. Scale bar: 50 µm.

The anatomical analysis of soybean leaves (Figure 3) evidenced a dorsiventral structure, with mesophyll composed of two layers of palisade cells, spongy parenchyma, and the presence of intracellular spaces.

All anatomical traits were significantly influenced (*p* < 0.001) by B and LQ as main factors, as well as by their interaction (B × LQ), except for the spongy thickness and intercellular space percentage, which were unaffected by B application (Table 2).

The leaf thickness decreased (*p* < 0.01) as the B concentration increased compared to the control. Among B treatments, K-0.5% determined the development of seedlings with the thinnest (*p* < 0.05) palisade parenchyma (Table 2).

Seedlings developed under RB light regime were characterized by a thicker (*p* < 0.001) palisade tissue, irrespectively from B concentration. The plant growth under FS and RB increased (*p* < 0.001) the spongy tissue thickness when compared to the FL regime. Consistently, FS and even more RB light determined the development of thicker (*p* < 0.001) leaves compared to FL. Finally, RB light induced a consistent decrease (*p* < 0.01) of the percentage of intercellular spaces accompanied by a more compact mesophyll organization (Table 2, Figure 3).

The interaction B × LQ determined the development of thickest leaves in particular for H_2_O × FS and H_2_O × RB as well as for K-0.01% × RB. This latter combination also determined the highest palisade thickness. The interactions H_2_O × RB and K-0.5% × FL produced in seedlings the most significant IS reduction.

#### 2.3.2. Pigments, Nitrogen Balance Index, and PSII Photochemistry

The effects of biostimulant and light quality and their interaction on pigments and functional traits of soybean seedlings were shown in Table 3.

As the main factor, the B application showed a significant effect (*p* < 0.05) on chlorophyll content. On the other hand, LQ as the main factor or in combination with biostimulant (B × LQ) determined significant changes (*p* < 0.001) on chlorophylls, flavonoids, and anthocyanins (Table 3). More specifically, the concentration K-0.5% reduced (*p* < 0.05) chlorophyll content compared to control, K-0.01%, and K-0.05% (Table 3).

The applied LQ regimes differently modulated the leaf pigment composition. Namely, FS and RB increased (*p* < 0.05) seedling chlorophylls compared to FL light, while FS reduced (*p* < 0.05) the anthocyanin amount compared to FL and RB regimes. On the other hand, RB enhanced (*p* < 0.001) flavonoid leaf concentration compared to FL and FS regimes (Table 3).

The most significant interactions were K-0.01 × FL and K-0.5% × FL, which negatively affected the seedling chlorophyll content.

LQ significantly affected (*p* < 0.001) nitrogen balance index (NBI) alone or in combination with biostimulant (B × LQ). In particular, RB was the only light regime inducing a decline (*p* < 0.05) of NBI (Table 3).

The effective quantum yield (Φ_PSII_) and the non-photochemical quenching (NPQ) were significantly influenced (*p* < 0.001) by B and LQ as main factors, as well as by their interaction (B × LQ). In contrast, the maximum PSII photochemical efficiency F_v_/F_m_ was affected only by B and B × LQ interaction (*p* < 0.05) (Table 3).

The K-0.05% concentration determined the highest (*p* < 0.001) Φ_PSII_ and F_v_/F_m_, and the lowest (*p* < 0.001) NPQ values. Regardless of B concentration, Φ_PSII_ significantly increased (*p* < 0.001) in FS and RB compared to FL seedlings (Table 3). Conversely, NPQ decreased (*p* < 0.001) under FS and even more under RB compared to FL light regime (Table 3).

All B × LQ interactions for functional traits were significant (Table 3). In particular, K-0.05% × FL significantly increased the NBI compared to H_2_O × RB and K-0.05% × RB. Moreover, K-0.5% × FL determined the highest NPQ, whereas K-0.05% × FL interaction promoted the highest Φ_PSII_ value within the FL regime. K-0.05% × FL produced a significant increase in F_v_/F_m_ compared to H_2_O × FL.

## 3. Discussion

This study evaluated for the first time the interaction between B application and LQ on *Glycine max* L. Merrill, a species widely consumed around the world as a source of protein-rich foods and beverages. The seed pre-treatment with the amino acid-based biostimulant Kaishi and the LQ regime during plant growth, as single factors or in interaction, strongly influenced the bioactive compound synthesis in sprouts producing an enrichment of antioxidant capacity, protein, and carbohydrate amount compared to the controls. On the other hand, during the seedling development, the growth under different LQ regimes acted as the main factor in modifying some leaf functional and anatomical traits influencing the seedling photosynthetic behaviour.

### 3.1. Effects of Biostimulant Seed Pre-Treatment and Light Quality on Sprout Bioactive Compounds

In the last decades, modern agricultural practices have emphasised the use of biostimulants to improve crop yield in a sustainable way. However, the observed effects strongly depend on species, kind of biostimulant, and application method [30,31,32,33,34,35].

The pre-treatment with different agents generally decreases seed dormancy and improves the metabolic processes occurring before radicle emergence. In particular, protein hydrolysates may modify the number of amino acids stored into the seeds as nutrients and energetic reserve, affecting germination and plant development [36,37,38,39,40,41,42].

Conversely to these findings, the amino acid-based biostimulant used in our study did not promote germination or days to 50% emergence compared to control. We suppose that the specific doses used for seed pre-treatment did not satisfy the seed metabolic activity for the germination process. On the other hand, the pre-treatment proved effective after germination since it enhanced the sprout nutritional traits and plant development in combination with specific LQ regimes.

However, we cannot outline a B dose-dependent trend for nutraceutical compounds because, in all treated samples, the bioactive molecule amount (e.g., antioxidants, chlorophylls, carotenoids) and carbohydrate and protein content were higher than control. K-0.05% seemed to be the most appropriate concentration to obtain healthier sprouts. Our results were in part consistent with Kim et al. [43], who demonstrated that seeds soaked with increasing concentrations of persimmon fruit powder produced sprouts proportionally richer in amino acids, ascorbic acid, and polyphenols. The amino acid-based biostimulants promoted polyphenol production [44] by stimulating nitrogen metabolism enzymes involved in the synthesis of these compounds [45]. In our study, the growth of soybean sprouts under specific LQ regimes (RB and FS) enhanced the positive effect of B on phytochemicals compared to FL and continuous darkness.

Chlorophylls and carotenoids were stimulated under FS and RB, suggesting the crucial role of red and blue wavelengths in the synthesis of photosynthetic pigments [46,47], while the FL regime produced sprouts richer in proteins than the other light regimes. Consistent with Mastropasqua et al. [48], our data demonstrated that soluble proteins increased in sprouts grown under light compared to the dark and identified the FL light regime as the most effective in inducing the protein synthesis into greening cotyledons. Even if the highest protein content was found in K-0.5% × FL sprouts, the concentration of K-0.05%, increasing the soluble proteins under all light regimes compared to control and K-0.01%, evidenced a positive interplay between this specific concentration and light quality. Among antioxidants, the AsA content raised under FS and RB light regimes, especially when joined with K-0.05 and K-0.5%. The beneficial effect of these concentrations on seed metabolism was probably helped the increasing percentage of blue wavelength in the FS and RB light regimes (respectively 37% and 40%) compared to FL (12%). Consistent with this hypothesis, previous studies demonstrated that blue light promoted the expression of genes involved in the modulation of the ascorbic acid [48,49,50]. LQ regimes also alter the metabolism of phenolic compounds, which are generally more abundant in light- than in dark-grown sprouts [43,46,49]. The higher percentages of red and blue light may have stimulated soybean polyphenol synthesis as observed in several crop species [48], enhancing the expression of several related genes [51].

However, big differences may occur depending on plant species or degree of light exposure. Our data indicated that the interactions K-0.01, K-0.05, and K-0.5% × FS and RB were the most effective in favouring the polyphenol production in soybean sprouts.

The increased amount of AsA, polyphenol, and pigment content contributed to the high antioxidant capacity observed in FS and RB compared to the dark and FL sprouts at all B concentrations. These results highlighted that the interplay of B × LQ is a powerful means to obtain higher quality food.

Finally, soybean sprouts developed under dark generally contained more carbohydrates than those exposed to light, regardless of the LQ regime [48,49]. During germination, especially in photosynthetically active sprouts exposed to light, lipids, proteins, and carbohydrates are metabolized to gain energy for growth and several biological functions [48,49,52]. In our study, none of the LQ regimes affected the carbohydrate content compared to dark. Conversely, B at all tested concentrations increased the carbohydrate content compared to control, with the highest stimulation at K-0.05%. We assumed that the short period of light exposure (in our case, four days) was not adequate to induce mobilization of carbohydrates in soybean sprouts when photosynthesis was not yet started.

### 3.2. Effects of Biostimulant Seed Pre-Treatment and Light Quality on Photosynthesis and Early Plant Development

Previous research carried out on soybean plants demonstrated that seed treatment with different concentrations of fish-derived PHs positively affected the plant’s vital processes, increasing plant biomass, phenolic compounds, and chlorophyll [53]. The seed pre-treatment with Kaishi biostimulant did not affect plant biomass or morphological traits, except SLA. The specific concentration of K-0.05% inducing the highest SLA, under all LQ regimes, appeared the most appropriate to improve plant productivity [54].

Our data proved that the effect of LQ on leaf morphological traits was stronger than those of the B seed pre-treatments. The growth of seedlings under FS and RB reduced the total leaf area and shoot/root biomass ratio but increased SLA compared FL light regime, indicating the development of the smallest plants with a higher investment in leaves and roots biomass. This aspect may be physiologically advantageous for plants because the higher SLA implicates higher photosynthetic yield, while a more developed root system may favor the plant water and nutrient supply. Our results were consistent with other studies reporting the efficiency of RB light in inducing higher plant yields, dwarf growth, and root expansion [13,55,56,57,58].

Considerable changes in leaf anatomical characteristics occurred in soybean plants subjected to B treatments. In Paradiso et al. [59], the seed inoculation with plant-growth-promoting microorganisms (PGPMs) determined plants with thicker leaves characterized by larger intercellular spaces. These anatomical traits significantly improved the PSII photochemical efficiency resulting in more efficient photosynthesis and growth. In our study, the seed pre-treatment with Kaishi decreased the leaf thickness. The different response may be likely due to the diverse origin of the applied biostimulant: in the first case, a mixture of PGPMs, in our study, a protein hydrolysate. However, the seed pre-treatment with K-0.05%, consistent with higher SLA, improved the PSII photochemistry in soybean seedlings compared to control and other treatments, resulting in an investment of the absorbed light in photochemical reactions (higher Φ_PSII_ and F_v_/F_m_) rather than in photoprotective processes (lower NPQ).

The growth under FS and RB increased the photosynthetic efficiency and reduced the need for thermal dissipation processes [60] compared to the FL regime. This result may be ascribed to the higher red: blue ratio of FS and RB regimes than FL, which positively affected the photosynthetic apparatus [61]. It could also be suggested that the better photosynthetic efficiency in FS and RB seedlings may be due to the anatomical modifications induced by red and blue wavelengths preferentially absorbed in the upper leaf tissues [12,13,19,62]. According to previous findings, higher proportions of red and blue light in FS and RB regimes have led to the thickening of palisade and spongy tissues resulting in denser leaves than those of plants grown under FL light [63]. Leaf thickness significantly influences the space availability for chloroplast development [64]. Indeed, a denser palisade tissue generally contains more chloroplasts and chlorophylls involved in light-harvesting and photochemical reactions [16,65,66], improving the photosynthetic efficiency.

Even if the seed pre-treatment with Kaishi biostimulant did not produce any effects on pigments, the FS and RB light regimes differently modulated chlorophylls, anthocyanins and flavonoids, engaged in leaf photoprotection [7,14,67]. The RB light regime stimulated flavonoid synthesis. Following our results, it has been demonstrated that the RB LED regime enhanced the expression level of flavonoid-related genes compared to fluorescent light, leading to the increasing of these compounds [68].

The anthocyanin level decreased under FS compared to FL and RB leaves. It is noteworthy that anthocyanin production is activated by blue light and UV and in some species is augmented by far-red addition. We supposed that the effect of the high percentage of blue light inducing anthocyanin accumulation in RB leaves might be slowed down by the presence of green wavelength in FS leaves [69]. The anthocyanin level comparable between FL and RB was probably due to the different proportions among light spectrum wavelengths. This aspect needs to be further clarified.

Interestingly, the NBI decrease in RB seedlings compared to FL and FS indicated that the high flavonoid production needed a high carbon demand to produce carbon-based secondary compounds. This result suggested the occurrence of a significant trade-off between secondary and primary metabolism under RB light regimes [70,71,72,73].

Overall, our results indicated that seed pre-treatment with biostimulant Kaishi exerted positive outcomes on soybean, especially when combined with specific light growth regimes. The seed pre-treatment was not helpful for the germination process but alone or joined with LQ regimes significantly improved bioactive compounds in sprouts. The interplay between K-0.05% × FS and K-0.05% × RB favoured important physiological traits such as higher SLA and PSII photosynthetic efficiency linked to plant productivity.

The heatmap separated the controls (H_2_O) from most of the sprouts pre-treated with Kaishi (B) and all dark (D) sprouts from most of the sprouts exposed to the light quality regimes (LQ), indicating that both biostimulant and light have a significant role during sprouting. The best interactions B × LQ were K-0.05% × FS and K-0.05% × RB since they displayed the highest bioactive compounds’ content. Concerning the seedlings, the heatmap visualization showed that only K-0.05% greatly influenced the physiological and morphological traits regardless of the specific LQ regime. The seed pre-treatment with different B concentrations was particularly useful under the FL regime, which separated control from biostimulant-treated seedlings. Most of the differences were lost under FS and RB light regimes, suggesting that the biostimulant effect was less critical than LQ in inducing changes in plant structure and function. Moreover, the interaction B × LQ significantly affected leaf anatomy and pigment content in seedlings, with positive implications on the photosynthetic process.

## 4. Materials and Methods

### 4.1. Seed Pre-Treatment and Germination

The biostimulant (Kaishi, AMM n°1171296) used in this study is manufactured and distributed by Sumi Agro France (251 rue de Faubourg Saint Martin, 75010 Paris, France, www.sumiagro.fr (accessed on 9 March 2018). Kaishi is a biostimulant with a unique liquid formula containing L-amino-acids of vegetal origin extracted through an enzymatic hydrolysis process and applied in biological agriculture.

Dry soybean seeds (*Glycine max* L. Merrill, Bulgarian variety) were soaked for 4 h in biostimulant solutions with different concentrations: 0.01%, 0.05%, and 0.5% (the following abbreviations were used throughout the whole text K-0.01%, K-0.05%, K-0.5%). Distilled water (H_2_O) served as a control.

Solutions were prepared by adding to the biostimulant (liquid formula) different amounts of distilled water necessary to obtain the desired concentrations (*v/v*). Each seed was soaked in 1 mL of solution for 4 h. After, seeds were carefully placed in Petri dishes supplied with a double layer of filter paper wetted with distilled water and incubated in the dark at 24 ± 2 °C. The double layer of filter paper was maintained wetted by adding distilled water when necessary.

The effect of the Kaishi treatment on germination was evaluated after 4 days of incubation in the dark when a constant count of germinated seeds was obtained. The germination percentage (G%) and the days to 50% emergence (E_50_), which indicates the rapidity in terms of days to obtain 50% germination, were calculated as reported in Noman et al. [74], using the following formulas:

G% = (Number of germinated seeds/Total number of seeds) × 100,(1)

E_50_ = t_i_ + (N/2 − n_i_)(t_j_ − t_i_)/(n_j_ − n_i_), (2)

where N = final number of germinated seeds; n_i_ = number of seeds emerged by count at time t_i_ when n_i_ < N/2; n_j_ = number of seeds emerged by count at time t_j_ when N/2 < n_j_. The germination test was performed on 50 seeds per biostimulant concentration for a total of 200 seeds and repeated four times.

### 4.2. Growth Conditions

**Figure 4:**
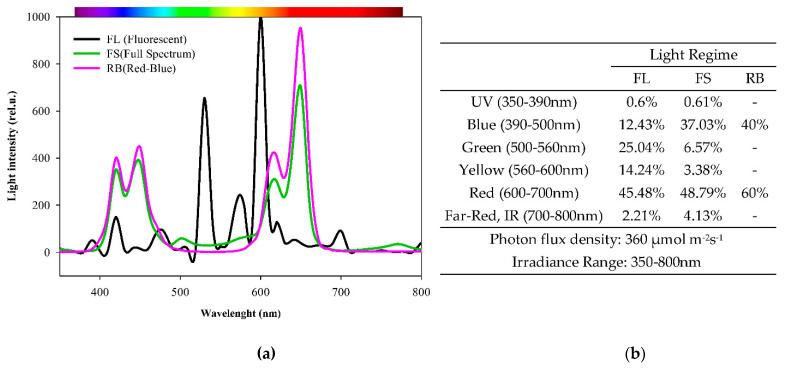
Light spectra used in the experiment (**a**). Spectral data and energy percentage of different light quality regimes. FL (white fluorescence tubes); FS (full-spectrum, LED); RB (red-blue, LED) (**b**).

For the light treatments, three growth chambers with different light quality regimes were used. The white fluorescent light (FL) was supplied by a combination of fluorescent tubes (Lumilux L36W/640 and L36W/830, Osram, München, Germany); full-spectrum (FS) was obtained by a combination of far-red, red, yellow, green, blue, UV-A and white light-emitting diodes (LEDs), and red-blue (RB, red 60%-blue 40%) derived from (LEDs) (LedMarket Ltd., Plovdiv, Bulgaria). The spectral composition of the light regimes was determined by an SR-3000A spectro-radiometer at 10 nm resolution (Macam Photometrics Ltd., Livingston, Scotland, U.K.), as reported in Figure 4. Sprouts and plants were grown under controlled conditions: light intensity of photosynthetic photon flux density (PPFD) 360 µmol photons m^−2^ s^−1^ for each light treatment, day/night air temperature 24/18 °C, relative air humidity 60–70%, and a photoperiod of 14 h.

At 4 DAS, 40 sprouts germinated from control (H_2_O) and biostimulant pre-treated seeds (K-0.01%, K-0.05%, and K-0.5%) were carefully placed in Petri dishes supplied with a double layer of filter paper wetted with distilled water. Then they (10 sprouts for each biostimulant concentration × light treatment) were moved to the growth chambers for further 4 days under dark (D), white fluorescent light (FL), full-spectrum (FS), and red-blue (RB). At 8 DAS, when they reached the size for the market demand, sprouts were collected for biochemical analyses.

A cohort of 15 germinated seeds from control (H_2_O) and each biostimulant concentration (K-0.01%, K-0.05%, and K-0.5%) was transplanted in plastic 1.0 L pots filled with tap water and left to grow until the achievement of the V1 stage (fully developed trifoliated leaves) under three light quality regimes: FL, FS, and RB (5 sprouts × light treatment).

The pots were refilled with tap water to field capacity when necessary. At 24 DAS, the seedlings were subjected to measurements of photosynthetic activity, leaf anatomy, and leaf functional attributes.

### 4.3. Analyses on Soybean Sprouts

Generally, sprouts used as food supplements are grown in total darkness [48]. Here, to assess the possible interaction B × LQ, the sprouts germinated from pre-treated seeds with increasing biostimulant concentrations (K-0.01%; K-0.05% and K-0.5%) were grown in continuous darkness (D), and also under the 3 different light quality regimes (FL, FS, RB).

The sampling for the biochemical analyses was carried out at 9:00 a.m. in the morning.

#### Biochemical Analyses

Biochemical analyses were carried out on 10 sprouts for each biostimulant concentration × light treatment. Each single sprout equates one replica.

The antioxidant capacity of soybean sprouts was determined by ferric reducing antioxidant power (FRAP) assay according to the method reported by George et al. [75], modified by Vitale et al. [19].

Briefly, samples (0.250 g) were ground in liquid nitrogen, mixed with 60:40 (*v/v*) methanol/water solution, and centrifuged at 14.000 rpm for 15 min (4 °C). FRAP reagents (300 mM acetate buffer pH 3.6; 10 mM tripyridyltriazine (TPTZ), 40 mM HCl and 12 mM FeCl_3_) were added to the extracts of each sample in 16.6:1.6:1.6 (*v/v*), respectively. After 1 h in darkness, the absorbance at 593 nm was measured with a spectrophotometer (UV-VIS Cary 100, Agilent Technologies, Palo Alto, CA, USA). Trolox (6-hydroxy-2,5,7,8-tetramethylchroman-2-carboxylic acid) was used as the standard, and total antioxidant capacity was quantified and expressed as µmol Trolox equivalents per mg of fresh weight (µmol TE g^−1^ FW).

Total polyphenols were determined as reported in Arena et al. [76]. Powdered samples (0.200 g) were extracted in methanol at 4 °C and centrifuged at 11.000 rpm for 5 min. Extracts were mixed with 1:1 (*v/v*) 10% Folin–Ciocâlteu reagent and, after 3 min, with 5:1 (*v/v*) 700 mM Na_2_CO_3_ solution. Samples were incubated for 2 h in darkness. Then, the absorbance at 765 nm was measured with a spectrophotometer (UV-VIS Cary 100, Agilent Technologies, Palo Alto, CA, USA). The total polyphenol content was calculated and expressed as mg of gallic acid equivalents per g of fresh weight (mg GAE g^−1^ FW) from the calibration curve using gallic acid as standard.

The ascorbic acid (AsA) content was determined using the Ascorbic Acid Assay Kit (MAK074, Sigma-Aldrich, St. Louis, MO, USA), following the procedure reported by Costanzo et al. [77]. Briefly, 10 mg of sample was homogenized in 4 volumes of cold AsA buffer and then centrifuged at 13.000 rpm for 10 min at 4 °C. The liquid fraction was mixed with AsA assay buffer to a final volume of 120 μL. The assay reaction was performed by adding the kit reagents to the samples. In this assay, the AsA concentration was determined by a coupled enzyme reaction, which develops a colorimetric (570 nm) product proportionate to the amount of ascorbic acid contained in the sample. The concentration of ascorbic acid in the samples was referred to as a standard curve and expressed in ng µL^−1^.

Total chlorophylls and carotenoids were determined according to Lichtenthaler [78]. Pigments were extracted from powered samples (0.200 g) in ice-cold 100% acetone and centrifuged at 5.000 rpm for 5 min (Labofuge GL, Heraeus Sepatech, Hanau, Germany). The absorbance of supernatants was measured by spectrophotometer (Cary 100 UV-VIS, Agilent Technologies, Santa Clara, CA, USA) at wavelengths of 470, 645, and 662 nm and pigment concentration expressed as mg per g^−1^ of fresh weight (mg g^−1^ FW).

Total carbohydrates content was determined following the anthrone method reported by Hedge and Hofreiter [79]. Briefly, powered samples (10 mg) were mixed with 2.5 N HCl in which carbohydrates are first hydrolyzed into simple sugars. The concentration was estimated by the anthrone reagent dissolved in ice-cold H_2_SO_4_. In a hot acid medium, glucose is dehydrated to hydroxymethyl furfural that forms with anthrone, a green-coloured product with an absorption maximum at 630 nm. The absorbance was measured by a spectrophotometer (UV-VIS Cary 100, Agilent Technologies, Palo Alto, CA, USA). The number of total carbohydrates was calculated using a glucose standard curve and expressed as mg glucose equivalents per g^−1^ of fresh weight (mg GE g^−1^ FW).

Total soluble protein content was determined according to Bradford [80] and Im et al. [81]. Powdered samples (0.200 g) were homogenized in 0.2 M potassium phosphate buffer (pH 7.8 + 0.1 mM EDTA). Samples were centrifuged at 10.000 rpm for 20 min at 4 °C. The supernatant was added to the dye reagent, and the absorbance was read at 595 nm using a spectrophotometer (UV-VIS Cary 100, Agilent Technologies, Palo Alto, CA, USA). The total soluble protein content was calculated from a calibration curve using bovine serum albumin (BSA) as standard and expressed as mg BSA equivalents per g^−1^ FW (mg BSA eq g^−1^ FW).

### 4.4. Analyses on Soybean Seedlings

Morphological parameters, leaf functional attributes, leaf anatomy determinations, and chlorophyll *a* fluorescence emissions analysis were carried out at 24 DAS on 15 seedlings for each biostimulant concentration and 20 seedlings for each light quality regime. One seedling equates one replica.

#### 4.4.1. Morphological Parameters and Leaf Functional Attributes

The total leaf area and the total plant length were measured by digital images analyzed by ImageJ software (Image Analysis Software, Rasband, NIH, Bethesda, Maryland, USA). The biomass of shoot and root was determined on dry weight bases after oven-drying the samples at 75 °C for 48 h.

The Specific leaf area (SLA) was estimated following Cornelissen et al. [82] as the ratio of leaf area to leaf dry mass (DW) and expressed in cm^2^ g^−1^ DW. Leaf area (LA) was measured using ImageJ software (Image Analysis Software, Rasband, NIH, Bethesda, Maryland, USA) and expressed in cm^2^.

The relative chlorophyll, flavonoid, and anthocyanin content, as well as the nitrogen balance index (NBI), were determined by a plant pigment meter (Dualex, Force-A, Paris, France) equipped with a leaf-clip sensor and expressed in relative units.

#### 4.4.2. Leaf Anatomy

The leaf anatomical analyses were performed by collecting leaf segments from each middle leaflet of the first fully expanded trifoliate leaves. After sampling, each segment was fixed in the fixative solution (40% formaldehyde/glacial acetic acid/50% ethanol, 5/5/90 *v/v/v*) at 4 °C and processed for inclusion according to the standard histological protocols for light microscopy [83].

After, tissue cross-sections of 3 µm thickness were stained with 0.025% Toluidine Blue in citrate buffer 0.1M, pH 4. All images were acquired by a light microscope (Axioskop Zeiss, Oberkochen, Germany) equipped with a digital camera (AxioCam MRc5, Zeiss, Oberkochen, Germany) using the same magnification (20×) and analysed through the AxioVision software (Carl Zeiss AG, White Plains, NY, USA) and the ImageJ software (Image Analysis Software, Rasband, NIH, Bethesda, Maryland, USA). More specifically, three fields of view and three different positions per image of each section were stored and used to determine the total leaf thickness (µm) and the thickness (µm) of the palisade and spongy tissues within the mesophyll. All measurements were carried out carefully, avoiding veins. Finally, the incidence of intercellular spaces was expressed as a percentage (%) of tissue occupied by intracellular spaces over a given surface considering three portions along the leaf lamina.

#### 4.4.3. Chlorophyll a Fluorescence Emission Analysis

Chlorophyll *a* fluorescence measurements were performed using the IMAGING-PAM M-series, chlorophyll fluorometer (Heinz Walz GmbH, Effeltrich, Germany). The minimum (F_0_) and maximum (F_m_) fluorescence were determined in 30-min dark-adapted seedlings and were used to calculate the maximum quantum yield of photosystem II (PSII) as:

F_v_/F_m_ = (F_m_ − F_0_)/F_m_. (3)

Then plants were exposed to actinic light (800 µmol photons m^−^^2^s^−^^1^) for 7 min and every 40 s saturating pulses (10,000 µmol photons m^−^^2^s^−^^1^) with duration 0.8 s were applied to determine the steady-state (F’) and maximum (F_m_’) fluorescence in light-adapted state. The effective quantum yield of PSII, Φ_PSII_, was determined as described in Genty et al. [84], by the formula:

Φ_PSII_ = (F_m_’ − F)/F_m_’, (4)

The non-photochemical quenching NPQ was calculated as indicated in Bilger and Björkman [85] as:

NPQ = (F_m_ − F_m_’)/F_m_’. (5)

### 4.5. Statistical Analysis

The overall parameters were visualized by a heatmap (heatmap function). The heatmap was plotted by using the ClustVis program package (https://biit.cs.ut.ee/clustvis/online (accessed on 16 April 2021)) and clustering both rows and columns with Euclidean distance and average linkage. In heatmaps, the numeric differences are evidenced by the colour scale: red and blue indicate increasing and decreasing values, respectively.

All data were analysed using the SigmaPlot 12 software (Jandel Scientific, San Rafael, CA, USA). A one-way ANOVA was performed on the dataset for seed germination.

The influence of the two different independent factors, namely biostimulant concentration (B) and light quality treatment (LQ), and their possible interaction were analyzed by two-way ANOVA. The Kolmogorov–Smirnov test was used to check the normality. The Student–Newman–Keuls (SNK) test was applied for all pairwise multiple comparison tests with a significance level of *p* < 0.05). Whenever the interaction between B and LQ was significant, data were subjected to one-way ANOVA and multiple comparison tests were performed with the SNK coefficient.

## 5. Conclusions

The seed pre-treatment with Kaishi biostimulant, the different light quality regimes, and their interaction significantly modified the bioactive compound level in soybean sprouts and morpho-anatomical traits and photosynthetic efficiency in seedlings. More specifically, while germination was unaffected, the seed pre-treatment increased the sprout antioxidant charge and the protein and carbohydrate content producing a richer food than control. The beneficial effects of biostimulant were improved in sprouts grown under FS and RB light regimes than FL and D, with the most critical effect at K-0.05% for both FS and RB light growth conditions. In seedlings, the effect of seed pre-treatment was evident only for the concentration of K-0.05%, which promoted higher SLA and PSII photochemical efficiency compared to control. Compared to FL, the positive effect of the biostimulant was enhanced in seedlings grown under FS and RB light regimes. The present study provides evidence that seed pre-treatment with Kaishi biostimulant and the plant growth under FS and RB regimes is a practical approach to obtain, in a sustainable way, sprouts with a more elevated nutritional value and seedling with high photosynthetic efficiency.

## Figures and Tables

**Table 1:** Analysis of variance and means comparison for bioactive compounds in soybean sprouts in response to different biostimulant (B) concentrations (K-0.01, K-0.05, and K-0.5%) and light quality (LQ) regimes (D, FL, FS, and RB) as well as under 16 different combinations of B × LQ. Different letters within each column indicate significant differences according to Student-Newman-Keuls multiple comparison tests (*p* < 0.05). Asterisks (*) represent the level of significance for main factors (B, LQ) and their interaction (B × LQ): NS-not significant; * *p* < 0.05; ** *p* < 0.01; *** *p* < 0.001. Seeds treated with H_2_O served as a control.

	Bioactive Compounds						
	TAC	TPC	CHL	CAR	CARB	AsA	SP
**B**							
H_2_O	1.48 b	0.77 c	0.33 b	0.034 a	59 c	7.5 c	55 c
K-0.01%	1.59 a	0.92 a	0.30 b	0.038 a	65 b	12 b	60 b
K-0.05%	1.62 a	0.95 a	0.44 a	0.037 a	82 a	15 a	72 a
K-0.5%	1.58 a	0.88 b	0.44 a	0.035 a	69 b	15 a	70 c
**LQ**							
D	1.40 b	0.62 c	0.21 c	0.028 c	67 a	9.7 c	59 c
FL	1.60 a	0.83 b	0.40 b	0.023 b	71 a	12 b	72 a
FS	1.64 a	1.02 a	0.45 a	0.042 a	68 a	14 a	63 b
RB	1.64 a	1.04 a	0.46 a	0.042 a	67 a	14 a	63 b
**Interaction**							
H_2_O × D	1.72 a	0.56 e	0.20 c	0.028 b	57 c	5.3 d	61 c
K-0.01% × D	1.47 bc	0.59 e	0.22 c	0.029 b	62 c	7.4 c	54 d
K-0.05% × D	1.41 bc	0.69 d	0.17 c	0.024 b	92 a	12 b	56 d
K-0.5% × D	1.02 d	0.64 e	0.23 c	0.031 b	60 c	14 b	64 c
H_2_O × FL	1.51 b	0.79 d	0.39 b	0.031 b	67 b	7.2 c	54 d
K-0.01% × FL	1.58 b	0.74 d	0.38 b	0.028 b	62 c	13 b	63 c
K-0.05% × FL	1.56 b	0.93 c	0.42 b	0.038 ab	80 b	14 b	81 b
K-0.5% × FL	1.74 a	0.85 c	0.40 b	0.033 b	75 b	14 b	89 a
H_2_O × FS	1.34 c	0.85 c	0.38 b	0.038 ab	57 c	8.8 c	51 d
K-0.01% × FS	1.65 a	1.11 b	0.31 b	0.048 a	69 b	13 b	60 c
K-0.05% × FS	1.74 a	1.11 b	0.56 a	0.043 a	77 b	16 a	76 b
K-0.5% × FS	1.77 a	1.02 b	0.56 a	0.038 ab	71 b	17 a	65 c
H_2_O × RB	1.35 c	0.88 c	0.35 b	0.039 ab	55 c	8.8 c	52 d
K-0.01% × RB	1.68 a	1.25 a	0.30 b	0.049 a	67 b	14 b	62 c
K-0.05% × RB	1.76 a	1.05 b	0.59 a	0.043 a	77 b	17 a	77 b
K-0.5% × RB	1.76 a	0.99 b	0.59 a	0.038 ab	69 b	17 a	63 c
**Significance**							
B	***	***	***	NS	***	***	***
LQ	***	***	***	***	NS	***	***
B × LQ	***	***	***	**	***	***	***

TAC: total antioxidant capacity (µmol TE g^−1^ FW); TPC: total polyphenol content (mg GAE g^−1^ FW); CHL: total chlorophylls (mg g^−1^ FW); CAR: total carotenoids (mg g^−1^ FW); CARB: total carbohydrates (mg GE g^−1^ FW); AsA: ascorbic acid (ng µL^−1^); SP: soluble proteins (mg BSA eq g^−1^ FW).

**Table 2:** Analysis of variance and means comparison for morphological parameters and anatomical traits in soybean seedlings in response to different biostimulant (B) concentrations (K-0.01, K-0.05, and K-0.5%) and light quality (LQ) regimes (FL, FS, and RB) as well as under 12 different combinations of B × LQ. Different letters within each column indicate significant differences according to Student-Newman-Keuls multiple comparison tests (*p* < 0.05). Asterisks represent the level of significance for main factors (B, LQ) and their interaction (B × LQ): NS-not significant; * *p* < 0.05; ** *p* < 0.01; *** *p* < 0.001. Seeds treated with H_2_O served as a control.

	Morphological Parameters					Anatomical Traits			
	TLA	SLA	Lenght	Biomass	S/R	LT	PT	ST	IS
**B**									
H_2_O	20 a	221 b	40 a	0.37 a	1.47 a	140 a	71 a	48 a	14 a
K-0.01%	22 a	215 b	38 a	0.37 a	1.67 a	134 b	74 a	45 a	15 a
K-0.05%	21 a	286 a	40 a	0.35 a	1.41 a	130 c	73 a	45 a	14 a
K-0.5%	23 a	199 b	36 a	0.35 a	1.37 a	127 c	68 b	45 a	14 a
**LQ**									
FL	25 a	214 b	38 a	0.37 a	1.72 a	124 c	69 b	41 b	14 a
FS	20 b	240 a	41 a	0.35 a	1.26 b	132 b	70 b	47 a	15 a
RB	19 b	237 a	37 a	0.36 a	1.45 b	143 a	76 a	49 a	13 b
**Interaction**									
H_2_O × FL	21 a	210 b	45 a	0.38 a	1.89 a	125 c	66 d	41 b	17 ab
K-0.01% × FL	24 a	198 b	40 a	0.37 a	1.73 a	124 c	67 d	42 b	20 ab
K-0.05% × FL	29 a	271 a	35 a	0.36 a	1.88 a	131 bc	82 b	41 b	14 b
K-0.5% × FL	27 a	179 c	34 a	0.35 a	1.41 a	117 d	62 e	39 b	10 c
H_2_O × FS	21 a	206 b	40 a	0.38 a	1.11 a	147 a	71 cd	51 a	17 ab
K-0.01% × FS	22 a	224 b	37 a	0.37 a	1.73 a	127 c	68 cd	43 b	11 bc
K-0.05% × FS	15 a	299 a	49 a	0.33 a	1.13 a	129 bc	70 cd	52 a	13 b
K-0.5% × FS	23 a	232 b	37 a	0.37 a	1.10 a	126 c	70 cd	44 b	20 a
H_2_O × RB	19 a	248 b	34 a	0.36 a	1.41 a	149 a	75 c	51 a	8.3 c
K-0.01% × RB	20 a	226 b	41 a	0.39 a	1.56 a	152 a	87 a	51 a	18 a
K-0.05% × RB	19 a	291 a	35 a	0.35 a	1.22 a	132 bc	70 cd	42 b	14 b
K-0.5% × RB	18 a	185 c	39 a	0.39 a	1.62 a	139 b	72 cd	54 a	12 b
**Significance**									
B	NS	***	NS	NS	NS	***	***	NS	NS
LQ	*	*	NS	NS	***	***	***	***	**
B × LQ	NS	*	NS	NS	NS	***	***	***	***

TLA: total leaf area (cm^2^); SLA: specific leaf area (cm^2^g^−1^); Length: total seedling length (cm); Biomass: total seedling biomass (g DW); S/R: shoot/root biomass allocation; LT: leaf thickness (µm); PT: palisade thickness (µm); SP: spongy thickness (µm); IS: intercellular spaces (%).

**Table 3:** Analysis of variance and means comparison for pigments and functional traits in soybean seedlings in response to different biostimulant (B) concentrations (K-0.01, K-0.05, and K-0.5%) and light quality (LQ) regimes (FL, FS, and RB) as well as under 12 different combinations of B × LQ. Different letters within each column indicate significant differences according to Student–Newman–Keuls multiple comparison tests (*p* < 0.05). Asterisks (*) represent the level of significance for main factors (B, LQ) and their interaction (B × LQ): NS—not significant; * *p* < 0.05; ** *p* < 0.01; *** *p* < 0.001. Seeds treated with H_2_O served as a control.

	Pigments			Functional Traits			
	CHL	FLAV	ANTH	NBI	Φ_PSII_	NPQ	F_v_/F_m_
**B**							
H_2_O	37 a	1.32 a	0.208 a	28 a	0.45 b	1.46 b	0.741 b
K-0.01%	36 a	1.28 a	0.207 a	29 a	0.44 b	1.29 c	0.748 b
K-0.05%	38 a	1.27 a	0.205 a	30 a	0.51 a	1.12 d	0.765 a
K-0.5%	34 b	1.29 a	0.211 a	27 a	0.41 c	1.59 a	0.750 b
**LQ**							
FL	34 b	1.23 b	0.210 a	29 a	0.36 b	1.74 a	0.753 a
FS	37 a	1.28 b	0.204 b	29 a	0.49 a	1.26 b	0.751 a
RB	37 a	1.38 a	0.211 a	26 b	0.51 a	1.10 c	0.747 a
**Interaction**							
H_2_O × FL	35 a	1.23 b	0.205 bc	29 ac	0.40 c	1.88 b	0.726 b
K-0.01% × FL	32 b	1.25 b	0.213 ab	26 bc	0.28 d	1.89 b	0.749 ab
K-0.05% × FL	40 a	1.15 b	0.198 c	35 a	0.51 ab	1.09 d	0.784 a
K-0.5% × FL	30 b	1.29 b	0.222 a	27 bc	0.26 d	2.11 a	0.754 ab
H_2_O × FS	40 a	1.32 ab	0.202 bc	31 ab	0.46 b	1.39 c	0.735 ab
K-0.01% × FS	37 a	1.28 ab	0.207 bc	29 ac	0.52 ab	1.02 d	0.743 ab
K-0.05% × FS	37 a	1.16 b	0.203 bc	33 ab	0.51 ab	1.09 d	0.773 ab
K-0.5% × FS	34 a	1.35 a	0.203 bc	28 bc	0.45 b	1.51 c	0.754 ab
H_2_O × RB	34 a	1.43 a	0.218 ab	24 c	0.50 ab	1.10 d	0.762 ab
K-0.01% × RB	40 a	1.33 ab	0.202 bc	30 ac	0.53 a	0.97 d	0.751 ab
K-0.05% × RB	33 a	1.49 a	0.216 ab	22 c	0.49 ab	1.19 d	0.735 ab
K-0.5% × RB	37 a	1.24 b	0.209 bc	30 ac	0.51 ab	1.15 d	0.741 ab
**Significance**							
B	*	NS	NS	NS	***	***	*
LQ	*	***	**	*	***	***	NS
B × LQ	***	***	***	***	***	***	*

CHL: chlorophylls (r.u); FLAV: flavonoids (r.u); ANTH: anthocyanins (r.u); NBI: nitrogen balance index; Φ_PSII_: effective quantum yield of PSII; NPQ: non-photochemical quenching; F_v_/F_m_: maximum PSII photochemical efficiency.

## Data Availability

The data supporting the findings of this study are available from the corresponding author (CA) upon reasonable request.
